# Small Cell Lung Cancer in Transition: Recent Advances in Biology, Treatment, and Precision Medicine

**DOI:** 10.3390/cancers18162547

**Published:** 2026-08-08

**Authors:** Supriya Peshin, Ehab Takrori, Mohammad Sajid Mithani, Deepthi Devagudi, Joseph H. Yazji, Ayse Gul Korkmaz, Adit Dharia, Waleed Maher Mohammad Tayyem, Shaas Ibrahim Qadoumi, Sakshi Singal

**Affiliations:** 1 Division of Geriatrics, Department of Internal Medicine, University of Arkansas for Medical Sciences, Little Rock, AR 72204, USA; 2 College of Medicine, Alfaisal University, Riyadh 11533, Saudi Arabia; etakrori@gmail.com (E.T.); mohammedsajid2014@gmail.com (M.S.M.); 3 Department of Hospital Medicine, Adventist Health Hanford, Hanford, CA 93230, USA; devagud@ah.org; 4 Department of Internal Medicine, HCA Florida Oak Hill Hospital, Brooksville, FL 34613, USA; drjyazji@gmail.com (J.H.Y.); mdayskorkmaz@gmail.com (A.G.K.); adit.dharia@louisville.edu (A.D.); 5 Faculty of Medicine, Jordan University of Science and Technology (JUST), Irbid 22110, Jordan; wmtayyem21@med.just.edu.jo; 6 College of Medicine, Misr University for Science and Technology, Giza 12566, Egypt; shaas-qadoumi@outlook.com; 7 Division of Hematology and Oncology, Department of Internal Medicine, East Tennessee State University, Johnson City, TN 37614, USA

**Keywords:** small cell lung cancer, chemoimmunotherapy, durvalumab, tarlatamab, DLL3, liquid biopsy, circulating tumor DNA, molecular subtypes, precision medicine, brain metastasis

## Abstract

Small cell lung cancer (SCLC) is a highly aggressive neuroendocrine malignancy associated with rapid proliferation, early metastatic spread, and poor long-term survival. Despite high initial responsiveness to chemotherapy and radiotherapy, most patients experience relapse, and therapeutic progress historically remained limited. Recent years, however, have seen meaningful advances that are beginning to reshape the management of SCLC. In extensive-stage disease, the incorporation of PD-L1 inhibitors into platinum-etoposide chemotherapy has established chemoimmunotherapy as the first-line standard, while underscoring the continued need for more durable disease control. In limited-stage SCLC, consolidation durvalumab following concurrent chemoradiotherapy has emerged as a practice-changing strategy with significant survival benefit. In relapsed disease, DLL3-targeted therapy has opened a new therapeutic avenue, with tarlatamab demonstrating clinically relevant efficacy in previously treated extensive-stage SCLC and becoming the first approved bispecific T-cell engager in this setting. Alongside these milestones, ongoing investigation is focused on maintenance strategies, novel targeted agents, biomarker development, molecular subtyping, and more effective integration of systemic therapy with radiation-based approaches. Nevertheless, resistance, toxicity management, central nervous system involvement, and the absence of validated predictive biomarkers remain major barriers to sustained progress. This narrative review examines recent advances in the biology and clinical management of SCLC, with emphasis on practice-changing developments, emerging therapeutic platforms, and future strategies aimed at improving outcomes in this historically difficult-to-treat disease.

## Simple Summary

Small cell lung cancer is one of the fastest-growing and most difficult lung cancers to treat. It often spreads early, responds well at first to chemotherapy and radiation, but usually returns and becomes resistant to treatment. This review summarizes recent advances in understanding the disease and improving patient care. The most important changes include adding immune-based treatment to chemotherapy for widespread disease, using durvalumab after combined chemotherapy and radiation for disease limited to the chest, and introducing tarlatamab for cancer that has returned after prior treatment. Tarlatamab works by helping immune cells recognize and attack cancer cells. Researchers are also studying blood-based tests that detect tumor material, molecular subtypes of the disease, and other markers that may help predict treatment response. Despite these advances, long-term survival remains poor, and challenges such as treatment resistance, spread to the brain, side effects, and the lack of reliable predictive tests remain. Continued research is needed to select treatments more precisely, detect relapse earlier, and achieve longer-lasting disease control for patients.

## 1. Introduction

Small cell lung cancer (SCLC) remains one of the most lethal thoracic malignancies and continues to pose a disproportionate clinical burden despite representing only a minority of lung cancers. In the United States, lung cancer remains the leading cause of cancer-related death, and SCLC accounts for approximately 13% of lung cancers. Although its incidence has declined in parallel with smoking reduction, survival outcomes remain poor, and population-level improvements have lagged behind those seen in other lung cancer subtypes [1].

The biologic and clinical behavior of SCLC explains much of this poor prognosis. SCLC is characterized by rapid tumor growth, short doubling time, early hematogenous dissemination, and a marked propensity for widespread metastatic spread, including to the central nervous system. At the same time, it is initially highly responsive to platinum-based chemotherapy and radiotherapy, with response rates that historically distinguished it from many other solid tumors. However, these responses are typically transient, and most patients ultimately experience treatment resistance and relapse, which remains the defining clinical challenge of the disease [2].

For decades, therapeutic progress in SCLC was limited, with platinum-etoposide-based regimens remaining the cornerstone of treatment across stages and relatively few successful attempts to alter the disease course. More broadly, the lung cancer treatment landscape has been reshaped by immunotherapy and targeted therapy over the past decade, underscoring how comparatively limited progress in SCLC had remained until only recently [3]. The introduction of immune checkpoint inhibitors into first-line treatment for extensive-stage SCLC represented an important step forward, with IMpower133 and CASPIAN establishing chemoimmunotherapy as a modern standard of care. Nevertheless, the magnitude of benefit has generally been incremental rather than transformative, and durable long-term disease control remains uncommon for most patients [4].

More recently, the field has entered a more dynamic phase. In limited-stage disease, consolidation durvalumab after concurrent chemoradiotherapy has improved both progression-free and overall survival, signaling a meaningful expansion of immunotherapy into earlier-stage SCLC. In parallel, DLL3 has emerged as the most clinically validated therapeutic target in relapsed SCLC. Tarlatamab, a DLL3-directed CD3 bispecific T-cell engager, first showed durable activity in heavily pretreated disease, then received FDA approval, and was subsequently confirmed in phase III testing to improve overall survival compared with chemotherapy in patients whose disease had progressed during or after platinum-based therapy. These developments have shifted relapse management from a largely chemotherapy-recycling paradigm toward mechanism-based therapeutic sequencing [5].

At the same time, translational research is beginning to reshape how SCLC may be monitored and stratified. Because tissue acquisition is often limited and validated predictive biomarkers remain scarce, liquid biopsy approaches have attracted substantial interest. Early studies suggest that circulating tumor DNA (ctDNA) dynamics may correlate with treatment response, survival outcomes, and risk stratification in SCLC, while more recent work in limited-stage disease supports a possible role for ctDNA in identifying patients at higher risk of progression after chemoradiotherapy and consolidation strategies. Although ctDNA-based molecular residual disease (MRD) assessment is not yet routine in SCLC, it is increasingly relevant to translational research, biomarker development, and future adaptive trial design [6].

Accordingly, the contemporary SCLC landscape is no longer defined solely by stage-based chemotherapy and radiotherapy, but by an evolving framework that increasingly incorporates immunotherapy, DLL3-directed therapies, maintenance strategies, molecular subtyping, and biomarker-informed investigation. Unlike reviews focused primarily on a single disease stage, therapeutic class, or biologic feature, the present review provides an integrated overview across limited-stage, extensive-stage, and relapsed or refractory SCLC. It brings together recent practice-changing treatments, advances in tumor biology, emerging biomarkers, liquid biopsy approaches, central nervous system management, and ongoing clinical trials. By linking these developments across the full disease continuum, this review aims to provide a clinically relevant perspective on how SCLC management is moving from predominantly empirical, stage-based treatment toward more biologically informed and individualized care.

## 2. Small Cell Lung Cancer Overview

SCLC is a highly aggressive neuroendocrine subtype of lung cancer that accounts for approximately 13% to 14% of lung cancers overall [7,8]. Although its incidence has declined over recent decades, SCLC continues to carry a substantial clinical burden because of its rapid growth kinetics, early metastatic dissemination, and poor long-term survival. Population-based data from the United States show that the proportion of SCLC among all newly diagnosed lung cancers decreased from 14.5% in 2000 to 11.8% in 2019, reflecting a sustained epidemiologic decline but only minimal improvement in outcomes over the same period [9].

The decline in SCLC incidence has largely been attributed to reductions in cigarette smoking, which remains the dominant risk factor for this disease [9,10]. Tobacco exposure is strongly linked to SCLC pathogenesis, and contemporary reviews estimate that most patients diagnosed with SCLC have a history of smoking. In addition to smoking, several non-tobacco exposures have also been associated with lung carcinogenesis and may contribute to SCLC risk, including residential radon exposure, asbestos, and air pollution. These findings underscore that although SCLC is classically regarded as the most smoking-associated lung cancer subtype, environmental and occupational factors may also play a contributory role in disease development [8,11].

At the molecular level, the hallmark pathogenesis of SCLC is driven by inactivation of the tumor suppressor genes TP53 and RB1, alterations that are considered central to tumor initiation and progression [12]. These molecular abnormalities contribute to the characteristic biologic behavior of SCLC, including rapid cell proliferation, short doubling time, early hematogenous spread, and a strong tendency to present with advanced disease. Clinically, SCLC is notable for its paradoxical behavior; it is often highly sensitive to initial chemotherapy and radiotherapy, yet these responses are usually short-lived, with relapse occurring in the majority of patients. This pattern of early response followed by rapid resistance remains one of the defining features of the disease and a major reason why long-term survival has historically remained poor [8].

The clinical presentation of SCLC is variable and depends on both intrathoracic disease burden and the presence of metastatic spread. Although a substantial proportion of patients may be asymptomatic at diagnosis, symptomatic patients most commonly present with respiratory complaints such as cough, dyspnea, and hemoptysis. Contemporary review data indicate that cough occurs in approximately 40% of cases, shortness of breath in 34%, and hemoptysis in 10%, while others present with symptoms related to metastatic disease such as pleuritic pain, bone pain, headache, or focal neurologic deficits. At diagnosis, brain metastases are already present in approximately 15% of patients, further highlighting the aggressive and early disseminating nature of this malignancy [13].

Despite decades of clinical use of platinum-based chemotherapy as the therapeutic backbone, the prognosis of SCLC remains among the worst of all solid tumors [14]. Most patients present with extensive-stage disease, and even among those who initially respond to treatment, durable remission is uncommon. Recent contemporary data report a 5-year overall survival ranging from approximately 12% to 30%, with outcomes varying substantially by stage, while broader historical estimates have commonly placed overall 5-year survival in the 5% to 10% range [13,14]. The persistently poor survival of SCLC reflects not only its aggressive natural history but also the longstanding absence of durable, biomarker-driven treatment strategies that have transformed outcomes in other thoracic malignancies.

## 3. Molecular and Tumor Biology Advances in SCLC

### 3.1. Genomic Landscape

SCLC is characterized by a relatively homogeneous core genomic framework superimposed on substantial biologic and phenotypic heterogeneity. The defining genomic event in most cases is near-universal inactivation of TP53 and RB1, which together drive loss of cell-cycle control, genomic instability, and lineage plasticity. Beyond these hallmark alterations, recurrent molecular abnormalities involve chromatin remodeling, epigenetic regulation, MYC-family amplification, NOTCH pathway dysregulation, and alterations affecting DNA damage response pathways. Unlike non-small cell lung cancer, however, SCLC rarely harbors targetable oncogenic drivers that can be readily matched to approved therapies, which has historically limited the development of precision treatment strategies. This stands in marked contrast to NSCLC, where multiple actionable oncogenic drivers have enabled a mature targeted-therapy framework and substantially expanded precision treatment options [15]. This discrepancy helps explain why SCLC remained therapeutically stagnant for decades despite major advances in thoracic oncology overall [16].

Although SCLC has often been treated as a single clinicopathologic entity, more recent molecular profiling studies have shown that it is better understood as a family of biologically distinct states rather than a fully uniform disease. These differences are not captured well by routine histopathology alone and instead emerge through transcriptomic, epigenetic, and lineage-based analyses. This has shifted the field from a simplistic view of SCLC as merely a rapidly proliferative neuroendocrine tumor toward a more nuanced model in which tumor behavior, therapeutic vulnerability, and interaction with the microenvironment may differ meaningfully across biologic subsets [17].

### 3.2. Transcriptional and Molecular Subtypes

One of the most important advances in SCLC biology has been the recognition of transcriptionally defined molecular subtypes. The most widely used framework classifies tumors according to dominant lineage-defining transcriptional programs, particularly ASCL1 (SCLC-A), NEUROD1 (SCLC-N), and POU2F3 (SCLC-P). A fourth group was initially described as YAP1-high; however, more recent studies increasingly recognize an inflamed or non-neuroendocrine subtype, termed SCLC-I, rather than considering YAP1 expression a consistently stable and independent lineage class. In general, SCLC-A and SCLC-N tumors retain stronger neuroendocrine features, whereas SCLC-P and SCLC-I tumors exhibit lower neuroendocrine differentiation and distinct biologic characteristics [17,18].

Importantly, these subtypes should not be regarded as mutually exclusive or permanently fixed categories. Different transcriptional states may coexist within the same tumor, producing substantial intratumoral heterogeneity. Individual tumor regions or cellular populations may express overlapping subtype-associated programs, and the dominant phenotype identified from a limited biopsy may not fully represent the entire disease. This heterogeneity may contribute to variable treatment sensitivity among tumor-cell populations and allow resistant clones to persist despite an initially favorable response [19].

Subtype identity may also evolve during disease progression and under treatment pressure. MYC activation, NOTCH signaling, and epigenetic reprogramming can promote transitions from highly neuroendocrine states toward less differentiated or inflamed phenotypes. Therefore, a tumor classified as SCLC-A or SCLC-N at diagnosis may acquire non-neuroendocrine characteristics during treatment or at relapse. Such transitions may alter immune-cell infiltration, antigen presentation, expression of surface targets, and sensitivity to systemic therapy, providing a potential mechanism for acquired treatment resistance [19].

The subtype framework also has potential therapeutic implications. SCLC-A tumors frequently demonstrate strong neuroendocrine differentiation and increased expression of targets such as DLL3, supporting the development of antigen-directed treatment strategies. SCLC-N tumors are often associated with MYC-related biology and may have therapeutic vulnerabilities distinct from ASCL1-dominant disease. SCLC-P represents a less neuroendocrine state characterized by a tuft-cell-like transcriptional program, whereas SCLC-I is associated with greater immune-cell infiltration, interferon-related signaling, and activation of immune-associated pathways. These features have raised the possibility that SCLC-I tumors may be more responsive to immunotherapy than highly neuroendocrine subtypes, although this association has not yet been validated for routine treatment selection [20,21].

From a clinical perspective, both intratumoral heterogeneity and subtype evolution limit the reliability of subtype assignment from a single pretreatment biopsy. Future subtype-guided strategies may therefore require longitudinal assessment using repeat tissue sampling, circulating tumor cells, circulating tumor DNA, or integrated tissue and blood-based approaches. Molecular subtyping is increasingly being incorporated into translational studies and clinical-trial design, but prospective validation, standardized assays, and clinically actionable treatment algorithms are still required before subtype-directed therapy can become part of routine practice.

### 3.3. Lineage Plasticity, Epigenetic Regulation, and Treatment Resistance

The transcriptional subtypes of SCLC should not be viewed as permanently fixed categories, but rather as dynamic cellular states that may change during tumor progression and in response to treatment. Experimental and clinical profiling studies suggest that SCLC cells can transition between neuroendocrine and non-neuroendocrine phenotypes through changes in lineage-defining transcriptional programs. MYC activation, NOTCH signaling, and suppression or re-expression of neuroendocrine transcription factors have been implicated in these transitions. Consequently, a tumor classified as ASCL1- or NEUROD1-dominant at diagnosis may acquire features of a less neuroendocrine or inflamed state under therapeutic pressure, potentially altering its sensitivity to chemotherapy, immunotherapy, and DLL3-directed treatment [18,19].

Epigenetic regulation appears to be a major driver of this plasticity. Alterations in chromatin-remodeling proteins, histone-modifying enzymes, and DNA-methylation programs can modify transcription without requiring new genetic mutations. These reversible changes may allow tumor cells to enter drug-tolerant states, suppress apoptotic signaling, alter antigen presentation, and reactivate developmental pathways that support survival. Epigenetic reprogramming may therefore explain why tumors with similar genomic alterations can show markedly different therapeutic responses and why resistance can develop rapidly despite substantial initial tumor regression [18,19].

Treatment resistance in SCLC likely results from both selection of pre-existing resistant populations and therapy-induced cellular adaptation. Platinum-based chemotherapy may eliminate highly proliferative, treatment-sensitive cells while permitting the survival and expansion of less differentiated or drug-tolerant populations. Similarly, immune checkpoint inhibition may favor the emergence of immune-excluded states or tumor populations with impaired antigen presentation. Lineage switching may also modify the expression of therapeutic targets such as DLL3, potentially affecting the activity of targeted agents over time. These observations highlight an important limitation of biomarker assessment based on a single pretreatment biopsy, which may not represent the biologic state of the tumor at relapse.

From a clinical perspective, lineage plasticity supports the development of longitudinal biomarker strategies, including serial circulating tumor DNA analysis, circulating tumor-cell characterization, and repeat tissue sampling when feasible. It also provides a rationale for combination and sequential treatment approaches that target both the dominant tumor population and the adaptive mechanisms responsible for resistance. However, the timing, direction, and clinical significance of subtype transitions remain incompletely defined, and prospective studies are needed before dynamic subtype assessment can guide routine treatment selection.

Recent reviews have further emphasized that therapeutic resistance in SCLC reflects the combined effects of transcriptional heterogeneity, lineage plasticity, epigenetic adaptation, and treatment-mediated selection of resistant cellular populations [22,23]. A clinically important example of lineage plasticity is histologic transformation from non-small cell lung cancer to SCLC, particularly following targeted therapy. Although transformed SCLC shares some morphologic and molecular features with de novo SCLC, it differs in its evolutionary origin, underlying molecular alterations, biologic behavior, and therapeutic considerations. Transformed SCLC therefore provides a clinically relevant model of treatment-associated lineage switching and acquired resistance but should not be considered equivalent to the conventional transcriptional subtypes of de novo SCLC [24].

### 3.4. Tumor Microenvironment and Immune Contexture

Compared with many other thoracic malignancies, SCLC has historically been viewed as an immunologically challenging tumor. Despite its high tumor mutational burden and strong association with tobacco exposure, SCLC often exhibits an immune milieu that is poorly inflamed, characterized by limited effective T-cell infiltration, impaired antigen presentation, and an immunosuppressive microenvironment. These features may help explain why immune checkpoint blockade, although beneficial in a subset of patients, has generally produced incremental rather than transformative gains. The tumor microenvironment in SCLC is increasingly recognized as biologically diverse, with immune activity varying across molecular subtypes and along the neuroendocrine to non-neuroendocrine spectrum [20].

The inflamed subtype concept has been particularly important in reframing immune biology in SCLC. Tumors classified as SCLC-I or inflamed/non-neuroendocrine may demonstrate greater immune-cell infiltration, interferon-related signaling, and increased expression of immune-associated pathways compared with more classically neuroendocrine tumors. By contrast, highly neuroendocrine ASCL1- or NEUROD1-dominant tumors may be more immune-excluded and less permissive to productive antitumor immunity. This growing appreciation of subtype-specific immune contexture has practical implications: it may partly account for heterogeneity in immunotherapy benefit and suggests that future strategies will likely require better biologic selection, rational combinations, and approaches that actively remodel the tumor microenvironment rather than relying on checkpoint inhibition alone [21].

### 3.5. DLL3 Biology as a Therapeutic Target

Among the biologic discoveries with the clearest translational impact in SCLC, delta-like ligand 3 (DLL3) has emerged as the most clinically validated therapeutic target. DLL3 is an inhibitory ligand in the NOTCH signaling pathway that is aberrantly expressed on the surface of many SCLC cells, while showing minimal to absent expression in most normal adult tissues. This tumor-selective expression profile makes DLL3 particularly attractive for targeted therapeutic development, because it provides a means of discriminating malignant neuroendocrine cells from normal tissue with a potentially favorable therapeutic window. DLL3 expression is especially enriched in neuroendocrine SCLC, particularly within ASCL1-driven tumors, although expression can be heterogeneous across and within tumors [25].

The importance of DLL3 lies not only in its expression pattern but also in how effectively it has translated into multiple therapeutic platforms. Antibody-drug conjugates, bispecific T-cell engagers, and chimeric antigen receptor T-cell approaches have all been developed against DLL3, reflecting its versatility as a target. Among these, the greatest clinical success to date has come from bispecific T-cell engager therapy, most notably tarlatamab, which has helped establish DLL3-directed treatment as a realistic and clinically meaningful strategy in relapsed SCLC. At the same time, growing recognition of intratumoral heterogeneity and variable DLL3 expression suggests that future work will need to refine biomarker selection, resistance mechanisms, and sequencing strategies. Thus, DLL3 represents not only a targetable antigen but also a broader proof of concept that biologically informed therapy in SCLC is achievable [26].

Taken together, advances in molecular profiling have fundamentally changed the biologic understanding of SCLC. Rather than a single uniform neuroendocrine malignancy, SCLC is now recognized as a heterogeneous and dynamic disease composed of distinct transcriptional states, variable immune ecosystems, and targetable surface antigens such as DLL3. These insights have not yet fully translated into routine precision oncology for most patients, but they are increasingly shaping therapeutic development, biomarker strategy, and the design of contemporary clinical trials [18].

## 4. Current Standard of Care

### 4.1. Limited-Stage SCLC

The management of LS-SCLC remains centered on curative-intent multimodality therapy. For most patients with stage IIB-IIIC disease who are fit for combined treatment, the standard approach is concurrent platinum-etoposide chemotherapy and thoracic radiotherapy, given early in the treatment course whenever feasible. This strategy remains the backbone of care because SCLC is highly radiosensitive and chemosensitive at presentation, and concurrent treatment offers the best chance of durable disease control in appropriately selected patients. For carefully selected patients with very early-stage, node-negative disease, surgical resection may be considered, usually followed by systemic therapy with or without mediastinal management depending on pathologic findings [27].

A major recent change in LS-SCLC has been the incorporation of durvalumab consolidation after concurrent chemoradiotherapy in patients without disease progression. The phase III ADRIATIC trial showed significant improvements in progression-free and overall survival with durvalumab compared with placebo after chemoradiotherapy, and NCCN educational materials based on Version 2.2026 list stage IIB-IIIC SCLC as ChemoRT followed by durvalumab consolidation. This has reshaped the post-chemoradiotherapy standard and represents one of the most important advances in LS-SCLC in many years [5].

Although prophylactic cranial irradiation and thoracic radiotherapy sequencing remain important components of LS-SCLC care, treatment decisions are increasingly individualized according to performance status, nodal burden, neurocognitive considerations, and access to MRI surveillance. Even so, the defining principle in limited-stage disease remains aggressive curative-intent combined-modality treatment, now followed by immunotherapy consolidation in eligible patients [28].

### 4.2. Extensive-Stage SCLC

For ES-SCLC, the first-line standard of care is platinum-etoposide combined with a PD-L1 inhibitor, most commonly atezolizumab or durvalumab. This modern standard was established by IMpower133, which showed improved overall and progression-free survival with atezolizumab plus carboplatin-etoposide versus chemotherapy alone, and by CASPIAN, which demonstrated an overall survival benefit with durvalumab plus platinum-etoposide versus platinum-etoposide alone. These trials marked the first meaningful frontline survival advances in ES-SCLC in decades and firmly established chemoimmunotherapy as the preferred first-line approach for eligible patients [4].

More recently, the frontline paradigm has begun to evolve further with the addition of maintenance lurbinectedin plus atezolizumab for patients whose disease has not progressed after induction therapy with atezolizumab, carboplatin, and etoposide. In October 2025, the FDA approved this maintenance strategy for adults with ES-SCLC in that post-induction setting, and NCCN Version 2.2026 educational materials note approval of lurbinectedin in combination with atezolizumab maintenance. Accordingly, current ES-SCLC management is no longer limited to induction chemoimmunotherapy alone but now includes a maintenance option for selected patients after first-line treatment [29].

Despite these advances, outcomes in ES-SCLC remain poor overall, and most patients ultimately relapse. As a result, contemporary management still requires balancing evidence-based first-line therapy with early planning for subsequent treatment, supportive care, brain imaging surveillance, and clinical trial consideration whenever appropriate [28].

### 4.3. Relapsed/Refractory Disease

Relapsed or refractory SCLC remains the most therapeutically difficult setting, and treatment selection is influenced by prior therapy, performance status, pace of progression, and the interval since platinum exposure. Historically, options in this space were limited, and median survival with second-line therapy was poor; NCCN educational materials summarize historical survival with second-line treatment at approximately 4 to 6 months. In patients with a longer chemotherapy-free interval, platinum rechallenge may still be considered, whereas those with early progression are generally treated with non-platinum options or enrolled in clinical trials when available [27].

The relapsed setting has changed substantially with the emergence of tarlatamab, a DLL3-directed CD3 bispecific T-cell engager. Tarlatamab is now a major treatment option for patients with ES-SCLC whose disease has progressed on or after platinum-based chemotherapy and represents a meaningful shift away from reliance on cytotoxic therapy alone [27].

**Figure 1:**
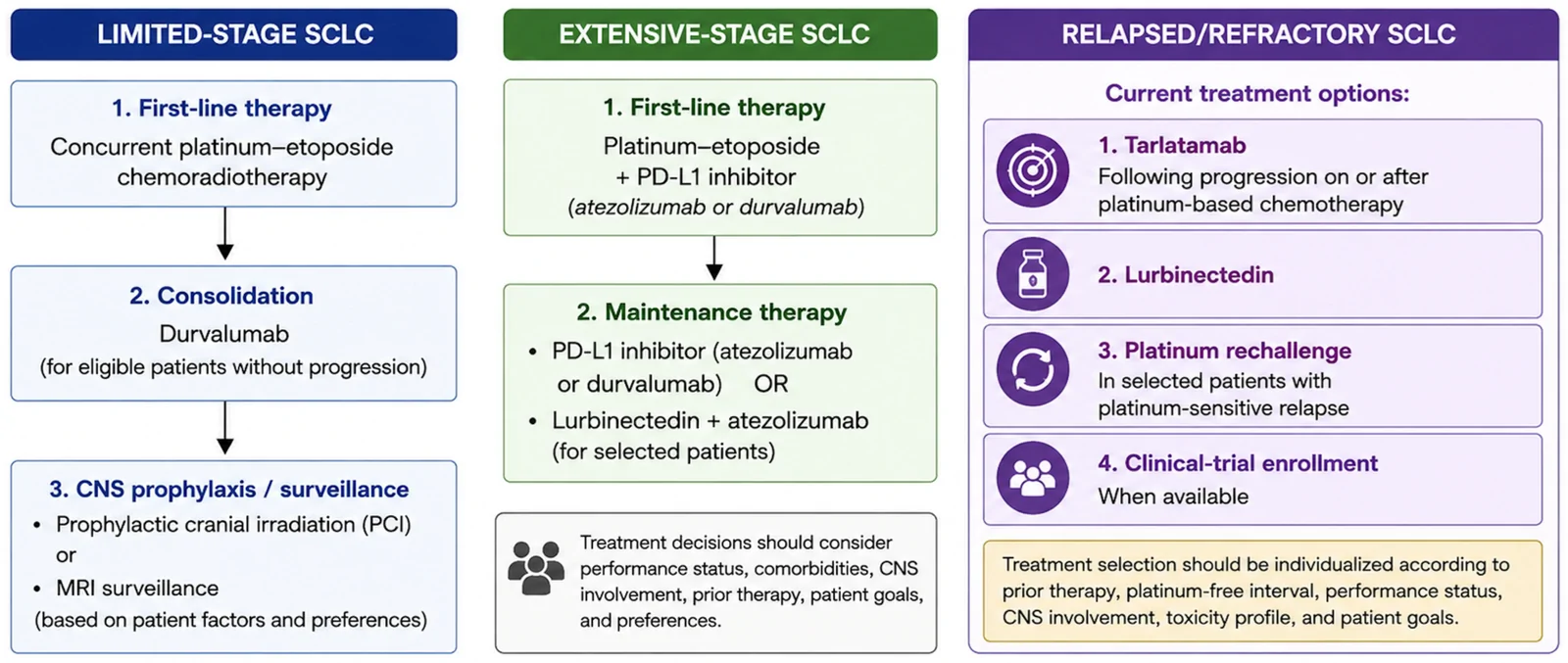
Contemporary treatment algorithm for small cell lung cancer (SCLC). Limited-stage SCLC is generally managed with concurrent platinum–etoposide chemoradiotherapy, followed by durvalumab consolidation in eligible patients without disease progression; prophylactic cranial irradiation or magnetic resonance imaging surveillance may subsequently be considered according to patient-specific factors. Extensive-stage SCLC is treated with platinum–etoposide plus a programmed death-ligand 1 inhibitor, followed by maintenance immunotherapy or, in selected patients, lurbinectedin plus atezolizumab. In relapsed or refractory SCLC, treatment options include tarlatamab following progression on or after platinum-based chemotherapy, lurbinectedin, platinum rechallenge in patients with platinum-sensitive relapse, and clinical-trial enrollment. Treatment selection should be individualized according to prior therapy, platinum-free interval, performance status, central nervous system involvement, toxicity profile, comorbidities, and patient goals. CNS, central nervous system; DLL3, delta-like ligand 3; MRI, magnetic resonance imaging; PCI, prophylactic cranial irradiation; PD-L1, programmed death-ligand 1; SCLC, small cell lung cancer.

Other active options remain relevant. Lurbinectedin continues to be an established therapy in relapsed SCLC, and topotecan remains a historical comparator and still-available treatment, although its toxicity profile and modest efficacy have limited enthusiasm in modern practice. Thus, the current relapsed/refractory landscape is increasingly defined by biologically targeted approaches, particularly DLL3-directed therapy, alongside selective use of chemotherapy rechallenges, lurbinectedin, and trial-based strategies [27]. An overview of the contemporary treatment pathway for limited-stage, extensive-stage, and relapse/refractory SCLC is shown in Figure 1 and summarized in Table 1.

## 5. Recent Therapeutic Advances in SCLC

### 5.1. Advances in First-Line Therapy for Extensive-Stage SCLC

The modern treatment era in ES-SCLC began with the incorporation of PD-L1 blockade into platinum-etoposide chemotherapy. IMpower133 established atezolizumab plus carboplatin-etoposide as a superior first-line approach compared with chemotherapy alone, and longer follow-up confirmed a sustained overall survival advantage with a tolerable safety profile. Similarly, the CASPIAN trial showed that durvalumab plus platinum-etoposide improved overall survival versus chemotherapy alone, with 3-year follow-up reinforcing this regimen as a durable frontline standard. Together, these trials transformed first-line treatment from chemotherapy alone to chemoimmunotherapy, but they also highlighted the continuing limitation that most patients still relapse and long-term survival remains uncommon [4].

### 5.2. Chemoimmunotherapy Era Refinement

Recent progress in ES-SCLC has focused less on replacing frontline chemoimmunotherapy than on refining it. One of the most important developments has been the emergence of maintenance intensification after induction treatment. In the phase III IMforte trial, patients without progression after atezolizumab, carboplatin, and etoposide had longer progression-free and overall survival with maintenance lurbinectedin plus atezolizumab than with atezolizumab alone, albeit with greater toxicity. This led to FDA approval in October 2025 for maintenance lurbinectedin in combination with atezolizumab after first-line induction therapy, making it one of the first clear examples of post-induction treatment refinement in ES-SCLC. These results suggest that the chemoimmunotherapy era is now evolving from a fixed induction-maintenance template toward a more adaptive strategy in which the post-induction period is treated as a therapeutic opportunity rather than simple observation or single-agent immunotherapy continuation [31].

### 5.3. Emerging First-Line Combinations Under Investigation

Several first-line or early-line combinations are now being explored to build on the modest gains of standard chemoimmunotherapy. Among the most notable are tarlatamab-based frontline maintenance and combination approaches. Early data from the phase Ib DeLLphi-303 program suggest that combining tarlatamab with anti-PD-L1 therapy in the first-line maintenance setting after chemoimmunotherapy is feasible and associated with encouraging survival signals, while other DeLLphi-303 cohorts have explored tarlatamab with concurrent first-line chemoimmunotherapy. Although these strategies remain investigational and are not yet standard of care, they are important because they reflect a broader shift in trial design: rather than waiting until late relapse, newer biologic agents are increasingly being moved earlier in the disease course [37].

### 5.4. Recent Advances in Relapsed/Refractory SCLC

Relapsed or refractory SCLC has historically been the setting with the greatest unmet need, characterized by brief responses to second-line therapy and poor median survival. Recent therapeutic progress in this space has been more substantial than in any prior period of SCLC drug development, with both cytotoxic and targeted approaches reshaping the post-platinum landscape. In particular, the field has moved beyond a nearly exclusive dependence on topotecan and platinum rechallenge toward a broader framework that includes lurbinectedin, DLL3-targeted therapy, and newer biomarker-informed investigational strategies [32].

### 5.5. Lurbinectedin and Post-Platinum Treatment Evolution

Lurbinectedin marked an important turning point in relapsed SCLC. In 2020, the FDA granted accelerated approval for lurbinectedin for adults with metastatic SCLC with disease progression on or after platinum-based chemotherapy, based on response-rate data that compared favorably with the historical performance of topotecan. Its approval was particularly notable because it represented the first new FDA-approved option in the immediate post-platinum setting in more than 20 years. Beyond its role as monotherapy, lurbinectedin has now also moved earlier into the treatment course through the IMforte maintenance strategy, reinforcing its growing importance across multiple disease settings. Thus, lurbinectedin has evolved from a second-line rescue option into a broader platform for treatment intensification and combination development in SCLC [32].

### 5.6. DLL3-Targeted Therapies

The most important biologically targeted advance in relapsed SCLC has been the successful clinical development of DLL3-targeted therapy. Tarlatamab, a DLL3-directed CD3 bispecific T-cell engager, received accelerated FDA approval in May 2024 for ES-SCLC progressing on or after platinum-based chemotherapy and traditional approval in November 2025 after confirmatory phase III data. In DeLLphi-304, tarlatamab significantly prolonged overall survival compared with chemotherapy in patients whose disease had progressed during or after platinum-based treatment, establishing DLL3 targeting as a clinically meaningful strategy rather than merely a promising translational concept. This was a landmark development for the field: it validated a surface antigen–directed approach in SCLC and shifted relapse management toward mechanism-based therapy [33].

### 5.7. Other Emerging Relapse Strategies

Beyond lurbinectedin and tarlatamab, the relapsed setting is increasingly populated by rational combination strategies, alternative DLL3-directed platforms, and biomarker-enriched clinical trials. Emerging therapeutic development increasingly focuses on biologically informed strategies, including cell-surface antigen-directed therapies, DNA-damage response targets, epigenetic regulators, and rational combinations designed to overcome subtype heterogeneity and adaptive treatment resistance [22,23]. Current investigational efforts include combining tarlatamab with checkpoint inhibition, integrating targeted agents earlier after relapse, and using biologic features such as molecular subtype, immune contexture, or circulating tumor DNA dynamics to better define which patients are most likely to benefit. Although many of these strategies remain in early-phase development, the important recent change is conceptual: relapse trials in SCLC are no longer dominated solely by nonselective chemotherapy comparisons but increasingly incorporate targeted, biomarker-informed, and immunologically based treatment designs.

### 5.8. Advances in Limited-Stage SCLC

**Figure 2:**
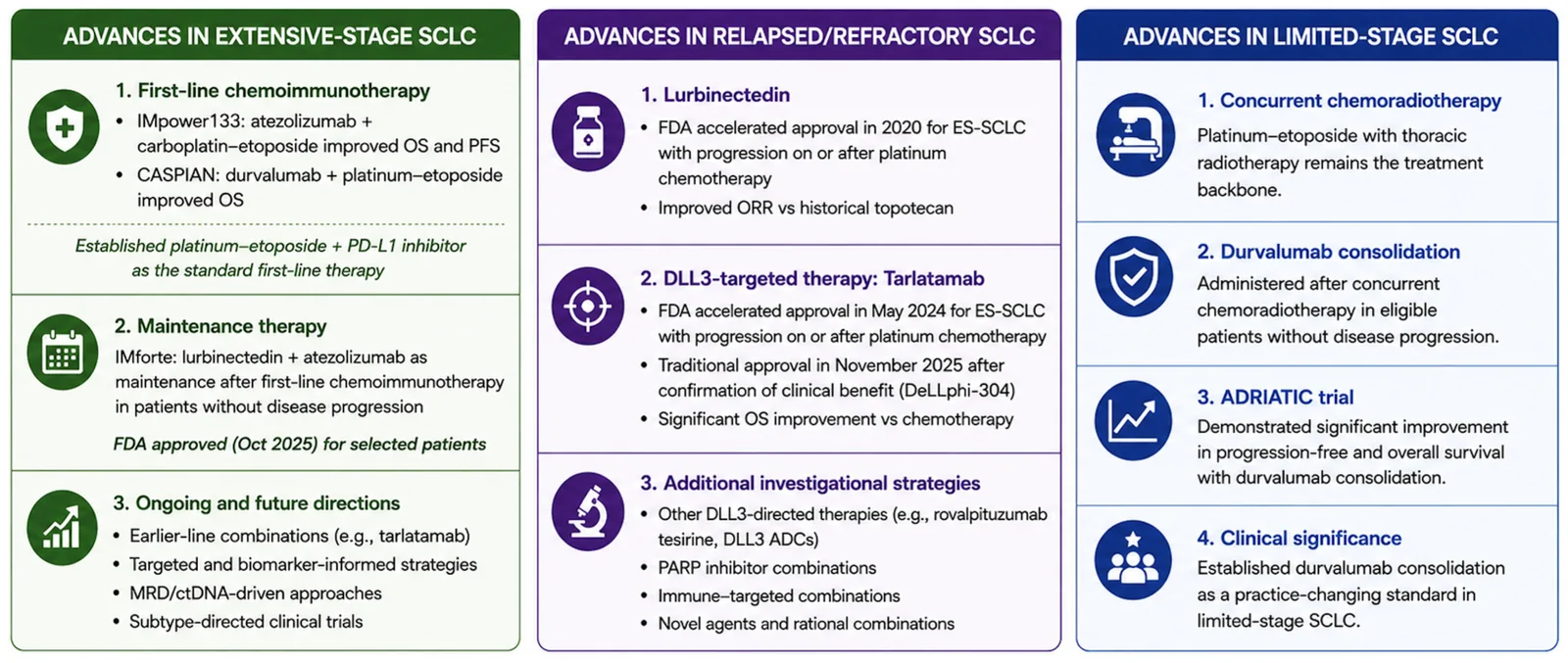
Timeline of major therapeutic advances in small cell lung cancer (SCLC). In extensive-stage SCLC, the IMpower133 and CASPIAN trials established platinum–etoposide plus PD-L1 inhibition as first-line therapy, while the IMforte trial supported maintenance lurbinectedin plus atezolizumab in selected patients without disease progression after induction chemoimmunotherapy. In relapsed or refractory disease, lurbinectedin expanded post-platinum treatment options, and DLL3-directed therapy with tarlatamab demonstrated an overall survival benefit over chemotherapy; additional DLL3-directed agents, PARP inhibitor combinations, immune-targeted approaches, and other rational combinations remain under investigation. In limited-stage SCLC, concurrent platinum–etoposide chemoradiotherapy remains the treatment backbone, and the ADRIATIC trial established durvalumab consolidation as a practice-changing standard for eligible patients without progression after chemoradiotherapy. ctDNA, circulating tumor DNA; DLL3, delta-like ligand 3; ES-SCLC, extensive-stage small cell lung cancer; MRD, molecular residual disease; ORR, objective response rate; OS, overall survival; PARP, poly(ADP-ribose) polymerase; PD-L1, programmed death-ligand 1; PFS, progression-free survival; SCLC, small cell lung cancer.

Limited-stage SCLC (LS-SCLC) has also seen a major therapeutic advance, as shown in Figure 2. For decades, concurrent platinum-based chemoradiotherapy was the standard backbone, with little change beyond optimization of radiation timing and use of prophylactic cranial irradiation. The phase III ADRIATIC trial altered this landscape by showing that durvalumab consolidation after concurrent chemoradiotherapy significantly improved both progression-free and overall survival compared with placebo in patients without progression after chemoradiotherapy. NCCN educational materials based on Version 2.2026 now incorporate this approach, reflecting a true change in routine practice. This finding is especially important because it extends meaningful immunotherapy benefit into earlier-stage SCLC and suggests that the post-chemoradiotherapy window may be a critical point for improving cure rates [5]. The principal clinical trials that have shaped recent treatment advances in limited-stage, extensive-stage, and relapsed SCLC are summarized in Table 2.

## 6. Biomarkers and Precision Medicine in SCLC

### 6.1. Why Biomarker Development Is Difficult in SCLC

Biomarker development in SCLC has been substantially more difficult than in non-small cell lung cancer because the biology and clinical course of SCLC create multiple barriers to stable, actionable biomarker discovery. Unlike NSCLC, SCLC rarely harbors recurrent targetable driver alterations that can be directly matched to approved therapies. Instead, it is defined by a relatively homogeneous core genomic backbone dominated by TP53 and RB1 loss, overlaid by marked transcriptional plasticity, intratumoral heterogeneity, and dynamic transitions between neuroendocrine and non-neuroendocrine states. This means that the biologic features most relevant to treatment response may not be fixed, and a single baseline tissue sample may fail to capture the evolving disease state [16].

A second major challenge is the practical difficulty of obtaining adequate tumor tissue. SCLC is frequently diagnosed using small bronchoscopic, needle, or cytology specimens, often from necrotic central tumors in clinically fragile patients. These samples are usually sufficient for diagnosis, but often inadequate for broad molecular profiling, serial testing, or robust immunohistochemical biomarker assessment such as PD-L1. Re-biopsy at progression is not routine in most patients, limiting the ability to study treatment-emergent resistance mechanisms or to adapt treatment according to biologic evolution [38].

Finally, SCLC tends to progress rapidly, so the clinical window for biomarker-driven decision-making is often narrow. Treatment usually starts quickly after diagnosis, and many patients deteriorate before more complex testing can realistically influence management. For all of these reasons, precision medicine in SCLC has advanced more slowly than in other thoracic malignancies despite major gains in biologic understanding [39].

### 6.2. Established Biomarkers: Current Limitations

At present, there are no validated biomarkers routinely used to select standard systemic therapy in SCLC, which remains one of the central limitations of the field. Although PD-L1 expression and tumor mutational burden (TMB) have been extensively studied in the context of immunotherapy, neither has emerged as a robust clinical decision tool in SCLC. PD-L1 expression is often low, variably measured, and difficult to assess reliably in the small and frequently crushed biopsy specimens typical of SCLC. In addition, even patients with low or absent PD-L1 expression may derive benefit from chemoimmunotherapy, limiting its discriminatory value [16].

TMB has also been biologically appealing because SCLC is strongly tobacco-associated and often genomically complex, but its clinical predictive value has been inconsistent. High TMB may enrich for immunotherapy responsiveness in some datasets, yet it has not demonstrated enough reproducibility or standardization to guide routine treatment selection. As a result, first-line chemoimmunotherapy and subsequent therapy choices are not currently assigned according to PD-L1 or TMB status in standard practice [16].

Even DLL3, while clearly important biologically and therapeutically, is not yet a universally required companion diagnostic for treatment assignment in the way that target biomarkers are used in NSCLC. DLL3 expression supports the rationale for targeted therapy and appears enriched in neuroendocrine SCLC, particularly ASCL1-driven tumors, but issues of heterogeneity, assay standardization, and threshold definition remain relevant. Thus, the established biomarker landscape in SCLC is notable less for what is firmly implemented than for the continuing absence of validated predictive tools [16].

### 6.3. Emerging Biomarkers

The most promising emerging biomarkers in SCLC fall into three broad groups: transcriptional subtype markers, liquid biopsy biomarkers, and candidate therapy-response markers. Molecular subtyping based on dominant transcription factor programs such as ASCL1, NEUROD1, POU2F3, and an inflamed/non-neuroendocrine state has become one of the most important conceptual advances in SCLC biology. These subtypes differ in neuroendocrine differentiation, immune infiltration, and therapeutic vulnerabilities, raising the possibility that future treatment selection may be guided by subtype rather than histology alone. However, this approach remains investigational and has not yet been standardized for routine clinical decision-making [20].

Liquid biopsy is another major area of momentum. Because tissue is limited and repeat sampling is uncommon, ctDNA and circulating tumor cells (CTCs) are especially attractive in SCLC. These platforms may enable noninvasive disease monitoring, molecular subtyping, prognostication, and earlier detection of relapse or resistance. ctDNA dynamics are being studied as markers of treatment response and molecular residual disease, while CTCs may allow evaluation of tumor-associated markers such as DLL3 and SLFN11. These approaches are promising because they can better capture disease heterogeneity and temporal evolution than a one-time tissue biopsy, although they still require further validation [38].

Among tissue- or blood-based candidate predictive markers, SLFN11 has attracted considerable interest as a potential biomarker of sensitivity to DNA-damaging therapy and PARP inhibitor-based strategies. Early studies suggest that higher SLFN11 expression may identify a subset with greater therapeutic vulnerability, but prospective validation is still needed before it can be incorporated into routine care. Other exploratory biomarkers under investigation include immune-related signatures, circulating microRNAs, extracellular vesicle-associated markers, and more complex integrated models that combine tumor, immune, and liquid biopsy features [39].

### 6.4. Practical Barriers to Biomarker Implementation

Even when promising biomarkers are identified, implementation in routine SCLC care faces substantial practical barriers. The first is specimen limitation: many patients are diagnosed on scant tissue, and the priority is often rapid confirmation of histology so treatment can start immediately. This leaves little material for extended biomarker panels and limits the feasibility of repeated testing over time [38].

The second barrier is assay standardization and validation. Many emerging SCLC biomarkers are currently measured using research assays, institution-specific thresholds, or exploratory platforms that are not harmonized across centers. Without standardized methodology, it is difficult to compare results across studies or to define clinically actionable cutoffs. This is particularly relevant for ctDNA, CTCs, subtype assignment, and DLL3-related testing [38].

A third barrier is clinical utility. For a biomarker to be useful in practice, it must change management in a way that is reproducible, timely, and accessible. In SCLC, even biologically compelling markers often do not yet have an approved, biomarker-matched treatment strategy attached to them. Cost, turnaround time, limited access to specialized testing, and the rapid tempo of clinical deterioration further reduce real-world uptake. Consequently, although biomarker science in SCLC is advancing quickly, implementation still lags behind discovery [16].

**Figure 3:**
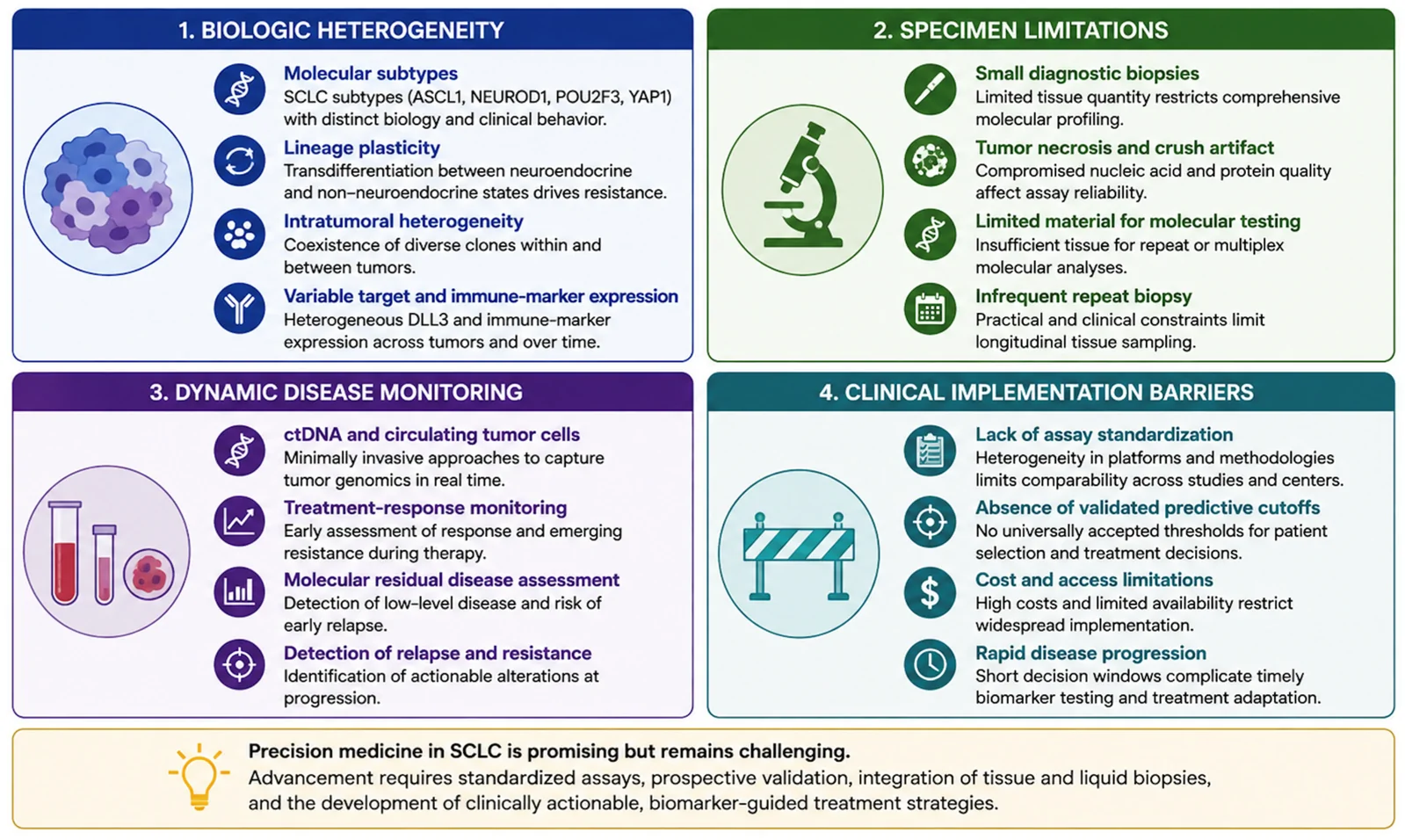
Biologic and practical challenges limiting biomarker development and implementation in small cell lung cancer (SCLC). Biologic barriers include the absence of common targetable oncogenic drivers, dynamic and heterogeneous transcriptional subtypes, an immune-excluded tumor microenvironment, and variable expression of therapeutic targets such as delta-like ligand 3. Practical barriers include limited tissue availability, rapid disease progression, short clinical decision windows, lack of standardized assays and thresholds, restricted access to specialized testing, and infrequent repeat biopsy. Consequently, no predictive biomarker is currently validated for routine systemic treatment selection in SCLC. Programmed death-ligand 1 expression and tumor mutational burden are not routinely used; delta-like ligand 3 is a clinically important therapeutic target but not a universal companion diagnostic; circulating tumor DNA and circulating tumor cells remain emerging approaches; schlafen 11 is investigational; and molecular subtypes are promising but not yet ready for routine clinical application. CTCs, circulating tumor cells; ctDNA, circulating tumor DNA; DLL3, delta-like ligand 3; PD-L1, programmed death-ligand 1; SLFN11, schlafen 11; TMB, tumor mutational burden.

In summary, precision medicine in SCLC remains an area of major promise but limited routine applicability. Biomarker development is hindered by scarce tissue, biologic plasticity, rapid disease kinetics, and the absence of stable targetable drivers. PD-L1 and TMB have not provided reliable clinical selection tools, whereas transcriptional subtypes, ctDNA, CTCs, DLL3-associated testing, and SLFN11 represent more promising but still emerging directions. The next phase of progress will likely depend not only on biomarker discovery, but also on assay standardization, prospective validation, and the integration of biomarkers into trial designs that can meaningfully alter treatment decisions [16]. The principal biologic and practical barriers limiting biomarker development and real-world implementation in SCLC are outlined in Figure 3 and summarized in Table 3.

## 7. CNS Disease and Evolving Brain Management

### 7.1. CNS Tropism in SCLC

Over 50% of patients with SCLC develop CNS metastases, usually within two years of diagnosis, with more than 10% of patients already having brain metastases at the time of initial diagnosis [41]. Radiologically, SCLC brain metastases present as multiple lesions, especially in the frontal lobe, parietal lobe and cerebellum and best identified with contrast-enhanced MRI [42].

A multi-staged process has been theorized which begins with local invasion, where upregulation of an enzyme called neuron-specific enolase (NSE), present on the surface of neuroendocrine cells, activates nerve growth factor receptor (NGFR) and Wnt/β-catenin signaling pathways. This allows cancer cells to enter lymphatic and blood circulation to metastasize [43]. Several factors have been identified as showing a positive association with an increased risk of brain metastases in patients with SCLC, including young age (<65), male sex, higher T stage (T ≥ 3), extensive stage disease and worse performance status (PS ≥ 2) [41].

### 7.2. PCI Versus MRI Surveillance

Prophylactic cranial irradiation (PCI), whole brain radiotherapy in patients without evidence of brain metastases, has historically been a standard component of SCLC management because of the disease’s propensity for occult and subsequent CNS metastasis. Earlier randomized trials, performed in the pre-MRI era, showed that PCI reduces the incidence of brain metastases and in some settings improves survival, particularly among patients who respond to initial therapy [44,45]. As a result, PCI became widely adopted in both limited-stage and extensive-stage SCLC.

In the modern era, however, the value of PCI has been increasingly questioned because routine contrast-enhanced brain MRI now allows more accurate baseline staging and earlier detection of asymptomatic CNS relapse. The Japanese randomized trial in extensive-stage SCLC, Takahashi T et al., which mandated MRI before enrollment and during follow-up, found that although PCI reduced the incidence of brain metastases, it did not improve overall survival compared with active MRI surveillance [46]. These findings challenged the universal use of PCI and shifted attention toward MRI-based monitoring as a less neurotoxic alternative [47].

### 7.3. Management of Brain Metastasis in Modern SCLC Care

Previously, whole-brain radiotherapy (WBRT) was the standard treatment modality for SCLC brain metastases because CNS metastases were associated with high rates of intracranial progression. In addition, SCLC has been considered radiosensitive, reinforcing the use of WBRT [48].

Over the last decade, as more robust research has been published, management has become targeted. The current management in the case of brain metastases secondary to SCLC is dependent on the disease stage. In patients with limited brain metastases, the first-line treatment is stereotactic radiosurgery (SRS), which preserves quality of life [48].

Current data suggest that SRS can provide meaningful local control for patients with SCLC, particularly those with a limited number of lesions, small intracranial tumor volume, and access to MRI surveillance. However, the main limitation of SRS in SCLC is not due to failure of treated lesions, but rather the risk of distant intracranial failure elsewhere in the brain. In this context, the success of SRS depends heavily on imaging follow-up and readiness to deliver additional salvage therapy when needed [49].

WBRT continues to retain an important role in SCLC, particularly in patients with diffuse brain metastases, rapidly progressive intracranial disease, or when SRS is not technically feasible. In these settings, WBRT provides comprehensive intracranial coverage and may achieve more rapid global disease control and symptom palliation. However, its use must be weighed against the well-recognized risk of neurocognitive toxicity, especially in patients with more favorable prognosis and longer expected survival [48].

## 8. Ongoing Trials and Future Directions

The recent progress achieved in SCLC has created momentum, but it has also clarified how much work remains. Even with the incorporation of chemoimmunotherapy in extensive-stage disease, durvalumab consolidation in limited-stage disease, and DLL3-targeted therapy in relapse, most patients still experience progression, and durable long-term control remains uncommon. As a result, the next phase of progress in SCLC will likely depend not only on introducing new drugs, but also on moving active agents earlier in the disease course, refining patient selection, improving biologic monitoring, and designing trials that account for the molecular plasticity of the disease [16,50].

A major focus of ongoing clinical investigation is the earlier integration of DLL3-targeted therapy, particularly tarlatamab, into frontline and maintenance strategies. The phase III trial NCT06211036 is evaluating tarlatamab plus durvalumab versus durvalumab alone as maintenance treatment after first-line platinum-etoposide plus durvalumab in extensive-stage SCLC, reflecting a direct attempt to intensify post-induction therapy after initial disease control [51]. In parallel, early-line combination studies such as NCT05361395 and NCT06830694 are assessing tarlatamab with PD-L1 inhibition, with or without carboplatin and etoposide, in treatment-naive SCLC [52,53]. Another frontline phase III study, NCT07005128, is comparing tarlatamab plus durvalumab, carboplatin, and etoposide with standard durvalumab, carboplatin, and etoposide, further signaling that DLL3-directed therapy is being actively moved from the relapsed setting into first-line disease [54].

Ongoing trials are also attempting to extend the benefit of targeted therapy to patient populations that are often underrepresented in pivotal studies. For example, NCT07203053 is evaluating tarlatamab in patients with extensive-stage SCLC and ECOG performance status 2, an especially relevant population in real-world SCLC practice given the frequency of functional decline at diagnosis and relapse. This remains a broader issue across lung cancer trials, where a recent systematic review found that patients with active untreated brain metastases and leptomeningeal disease continue to be excluded from most studies, highlighting the importance of designing future SCLC trials that better reflect real-world CNS disease burden. This reflects an important future direction for the field, where not only developing more active therapies but also ensuring that trial populations better reflect the patients seen in routine clinical care [55,56,57].

Beyond DLL3, future therapeutic development in SCLC is increasingly shaped by the recognition that the disease is heterogeneous, plastic, and biologically dynamic. Recent reviews emphasize that subtype switching, lineage plasticity, antigen modulation, and changes in immune contexture may all contribute to resistance. This suggests that single static biomarkers will likely be insufficient in many cases. Instead, future studies may need to incorporate longitudinal biologic profiling, including serial ctDNA, circulating tumor cells, and repeat tissue analysis where feasible, to identify evolving therapeutic vulnerabilities and mechanisms of escape during treatment [22,58,59].

Accordingly, one of the most important future directions in SCLC is the development of adaptive biomarker-informed trial designs. Because tissue is often limited and disease evolution is rapid, liquid biopsy approaches are particularly attractive. Reviews of SCLC biomarker development increasingly highlight ctDNA and circulating tumor cells as tools for response assessment, molecular residual disease detection, relapse prediction, and potentially subtype monitoring. Although these approaches are not yet ready for routine biomarker-driven treatment assignment, they may become increasingly important for trial stratification and for defining biologically informed treatment pivots during therapy [16].

Another key direction is the expansion of immunotherapy beyond current checkpoint inhibitor paradigms. In limited-stage disease, the success of durvalumab consolidation after chemoradiotherapy has opened the door to further investigation of immunotherapy in earlier and potentially curative settings, including concurrent, consolidation, and neoadjuvant approaches. Contemporary reviews of limited-stage SCLC immunotherapy suggest that the next frontier will be determining how best to sequence immunotherapy with thoracic radiation, how to optimize toxicity management, and whether earlier immune intervention can improve cure rates rather than simply delay progression [16,60].

At the same time, the field continues to explore combination strategies designed to deepen and prolong response. These include pairing immune therapies with targeted agents, integrating newer antibody-based platforms, and developing rational combinations intended to overcome resistance in molecularly defined subsets. Reviews published in 2025 emphasize that future gains will likely come from more mechanism-based strategies rather than further recycling of nonselective cytotoxic regimens alone. This shift is already visible in the design of contemporary relapse and frontline trials, which increasingly incorporate biologic targets, subtype-informed hypotheses, and translational endpoints [22,50,61].

Ultimately, the future of SCLC management will likely depend on four parallel advances: moving active therapies earlier, improving biologic stratification, monitoring disease dynamically, and designing trials that account for intratumoral heterogeneity and lineage plasticity. The recent emergence of DLL3-targeted therapy has shown that meaningful biologically informed progress is possible in SCLC. The challenge now is to translate this momentum into broader and more durable benefit across both limited-stage and extensive-stage disease [16,58].

## 9. Conclusions

SCLC remains one of the most aggressive solid malignancies, but recent advances—including chemoimmunotherapy in extensive-stage disease, durvalumab consolidation after chemoradiotherapy in limited-stage disease, and DLL3-directed therapy with tarlatamab in relapsed disease—have meaningfully changed its treatment landscape. Precision medicine is also becoming increasingly relevant through molecular subtyping, immune profiling, DLL3 and SLFN11 evaluation, and liquid biopsy approaches such as circulating tumor DNA and circulating tumor cells; however, these biomarkers are not yet sufficiently validated to guide routine treatment selection. Future progress will depend on prospective biomarker validation, standardized assays, longitudinal monitoring of tumor evolution, and adaptive clinical trials that account for intratumoral heterogeneity and lineage plasticity. Moving effective therapies into earlier disease settings, improving treatment sequencing and central nervous system management, and broadening clinical-trial eligibility may ultimately support more individualized treatment and more durable outcomes for patients with SCLC.

## Figures and Tables

**Table 1:** Current standard of care in small cell lung cancer (SCLC) by disease setting.

Disease Setting	Standard Treatment Approach	Key Recent Refinement	Important Clinical Notes
Limited-stage SCLC	Concurrent platinum-etoposide chemoradiotherapy [5,16]	Durvalumab consolidation after chemoradiotherapy in eligible patients [5,16]	Curative-intent treatment; PCI or MRI surveillance may be considered in selected patients [5,16]
Extensive-stage SCLC	Platinum-etoposide plus PD-L1 inhibitor [4,16,30]	Maintenance strategies are emerging in selected patients, particularly lurbinectedin plus atezolizumab after induction chemoimmunotherapy [16,31]	Most patients eventually relapse despite frontline benefit [4,16,30]
Relapsed/refractory SCLC	Tarlatamab, lurbinectedin, platinum rechallenge in selected patients, and clinical trials [16,32,33,34,35]	DLL3-targeted therapy has changed the relapse landscape [33,35,36]	Treatment choice depends on platinum sensitivity, performance status, prior therapies, and treatment goals [16,32,35]

**Table 2:** Major recent therapeutic advances in small cell lung cancer (SCLC).

Trial	Phase and Disease Setting	Intervention and Comparator	Key Outcomes	Clinical Significance
Impower133 [4]	Phase III; previously untreated extensive-stage SCLC	Atezolizumab plus carboplatin and etoposide versus carboplatin and etoposide alone	Addition of atezolizumab improved overall survival and progression-free survival, with sustained benefit on longer follow-up	Established platinum-etoposide plus PD-L1 inhibition as a first-line standard for extensive-stage SCLC
CASPIAN [30]	Phase III; previously untreated extensive-stage SCLC	Durvalumab plus platinum-etoposide versus platinum-etoposide alone	Durvalumab significantly improved overall survival, with continued benefit demonstrated at 3-year follow-up	Confirmed chemoimmunotherapy as the preferred first-line treatment approach
ADRIATIC [5]	Phase III; limited-stage SCLC without progression after concurrent chemoradiotherapy	Durvalumab consolidation versus placebo	Durvalumab significantly improved both progression-free survival and overall survival	Established durvalumab as a practice-changing post-chemoradiotherapy strategy in eligible patients
IMforte [31]	Phase III; extensive-stage SCLC without progression after induction atezolizumab, carboplatin, and etoposide	Maintenance lurbinectedin plus atezolizumab versus atezolizumab alone	Combination maintenance improved progression-free and overall survival but was associated with greater toxicity	Established maintenance intensification as a therapeutic option after first-line chemoimmunotherapy and supported regulatory approval
DeLLphi-304 [33,35]	Phase III; extensive-stage SCLC progression during or after platinum-based chemotherapy	Tarlatamab versus standard chemotherapy	Tarlatamab significantly prolonged overall survival compared with chemotherapy	Confirmed the clinical benefit of DLL3-directed therapy and supported traditional approval of tarlatamab in relapsed disease
DeLLphi-303 [37]	Phase Ib; first-line maintenance setting in extensive-stage SCLC	Tarlatamab combined with PD-L1 inhibition after induction chemoimmunotherapy	The combination was feasible and demonstrated encouraging early activity and survival signals	Provided clinical rationale for moving DLL3-directed treatment into earlier disease settings; the strategy remains investigational

**Table 3:** Biomarkers in small cell lung cancer (SCLC): current status and limitations.

Biomarker	Biomarker Type	Proposed Role	Current Status in SCLC	Major Limitation
PD-L1 [16,40]	Tissue immune marker	Predict response to immunotherapy [16,40]	Not routinely used for treatment selection	Low/variable expression and limited predictive value in SCLC
Tumor mutational burden [16,40]	Genomic biomarker	Predict immunotherapy benefit [16,40]	Investigational	Inconsistent predictive performance and lack of standardization
DLL3 [35,38,40]	Surface tumor antigen	Therapeutic target and potential predictive biomarker [36,39,40]	Clinically important target, especially in relapsed disease	Assay standardization and expression heterogeneity
ctDNA [33,35]	Liquid biopsy biomarker	Monitor response, detect relapse, assess MRD [38,40]	Emerging	Requires prospective validation and standardized assays
Circulating tumor cells [38,39]	Liquid biopsy biomarker	Disease monitoring and biologic characterization [38,39]	Emerging	Technical complexity and limited routine availability
SLFN11 [18,39,40]	Tissue or cell-based biomarker	Predict sensitivity to DNA-damaging therapy or PARP-related approaches [18,39,40]	Investigational	Not prospectively validated for routine care
Transcriptional subtypes (ASCL1, NEUROD1, POU2F3, inflamed/non-NE) [18,21]	Molecular subtype biomarker	Stratify biology and potential therapeutic vulnerabilities [18,21]	Promising but not yet routine	Dynamic plasticity and lack of standardized clinical implementation

## Data Availability

No new data were created or analyzed in this study. Data sharing is not applicable to this article.
